# Study of Deformation Behavior and Microstructural Evolution in Multiphase Steel

**DOI:** 10.3390/ma11112285

**Published:** 2018-11-15

**Authors:** Jun Lu, Hao Yu, Xiaoni Duan, Chenghao Song

**Affiliations:** School of Materials Science and Engineering, University of Science and Technology Beijing, No. 30 Xueyuan Road, Haidain District, Beijing 100083, China; b20160180@xs.ustb.edu.cn (J.L.); s20170303@xs.ustb.edu.cn (X.D.); songchenghao28@126.com (C.S.)

**Keywords:** tensile deformation, retained austenite, phase transformation, grain subdivision

## Abstract

In the present work, the tensile deformation characteristics of the high performance multiphase steel with complex microstructures are investigated. A mixture of ferrite, bainite, and 14.4 vol% retained austenite (RA) with an average grain size of less than 3 μm of the matrix is obtained after specific heat treatment. Tensile tests are performed with increasing strain, i.e., 0%, 5%, 10%, 15%, and 20%. Then X-ray diffraction, transmission electron microscope and electron backscatter diffraction are utilized to analyze the deformation-transformation behaviors of the complex microstructures. Phase transformation of the RA, which is controlled by its morphology and distribution, contributes to high strain hardening capacity of the steel. The blocky-type RA that locates in ferrite grain boundaries shows less stability and transforms easily at early deformation stage, while the film-like RA that distributes between bainitic ferrite shows higher stability and transforms continuously throughout plastic deformation. Moreover, the substructure formation by dislocation configuration in ferrite grains begins with randomly distributed dislocations and ends up with cellular structures, resulting in ferrite subdivision during deformation and also grain refinement strengthening. As a result, the experimental steel is reinforced not only by the martensite transformation of RA, but also ferrite refinement.

## 1. Introduction

In recent years, advanced high strength steels (AHSS) are increasingly used in the auto industry due to their excellent mechanical properties, which can achieve weight reduction, higher security and good formability for vehicle structures [1,2]. Transformation induced plasticity (TRIP) steel is among one of the most promising products applied to lightweight auto-material for energy saving and environmental protection. This kind of steel has complex microstructures that mainly consist of ferrite, bainite, martensite, and retained austenite (RA). Good combination of strength and ductility can be achieved in this steel, high uniform elongation and high formability are acquired through the strain induced martensite transformation of RA during plastic straining, which also contributes to good work hardening capacity and delayed softening [3]. For example, in the ultra-fine grained (UFG) steel produced by accumulative roll bonding (ARB) process, the TRIP phenomenon is introduced to improve its strain-hardening ability and overcome the very poor uniform elongation of the UFG steels [4,5]. It is widely recognized that good mechanical performance of the TRIP-assisted steel attributes to appropriate stability of RA, which is controlled by the volume fraction, distribution, morphology, and carbon content of RA [6,7,8,9,10,11].

In general, excellent performance of such multiphase steel owes to not only TRIP effect but also mutual interactions of its complex phases. It is researched that in the duplex lightweight steel, the continuous substructure formation leads to grain refinement in both austenite and ferrite [12]. The deformation mechanism in ferrite is mainly controlled by dislocation slip owing to its high stacking fault energy and various slip system [13]. Increase of dislocation density and the formation of substructures lead to ferrite grain refinement and accelerated work hardening, resulting in reinforcement of these steels [14]. As for TRIP-assisted steel that owns multiphase microstructures, ferrite and austenite in the matrix exhibit different deformation behavior. Good mechanical properties of the TRIP steel owe to the collaborative microstructural evolution, which is investigated in more detail in this work.

Microstructural investigations focusing on RA stability, evolution, strain, and stress distribution characteristics in TRIP-assisted steel have been widely reported by researchers [1,3,5]. In the present work, the collaborative microstructural evolution of RA and the bcc matrix in multiphase TRIP-assisted steel was investigated. Commercial TRIP-assisted steel is generally based on carbon-manganese-silicon steel with product of strength and elongation (PSE) lower than 30 GPa·% [15], higher strength level of 1100 MPa grade TRIP-aided steel exhibiting PSE over 30 GPa·% was obtained and studied in this work. The complex microstructural matrix with a soft ferrite phase, a strong bainitic phase, a suitable proportion of RA, and a number of vanadium precipitates were analyzed by detailed characterizations. In the tensile experiments with increasing strain, transformation kinetics of RA and substructures formation of ferrite were investigated to study deformation behavior and microstructure evolution of the steel.

## 2. Materials Preparation and Experimental Procedure

### 2.1. Materials Production and Heat Treatment

**Figure 1:**
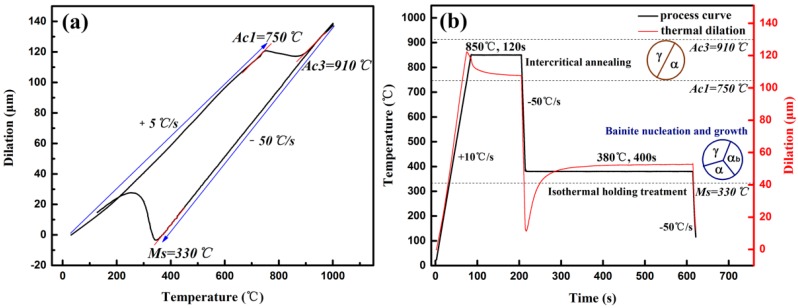
Schematic diagram of (**a**) phase transformation points and (**b**) optimal heat treatment condition carried out on a thermal dilatometer.

An alloy ingot with a nominal composition of Fe-0.20C-1.54Si-2.05Mn-0.21V (wt%) was prepared by vacuum melting in laboratory batch followed by die casting. The ingot was homogenized at about 1200 °C for 2 h and then hot rolled to 3.6 mm at a rolling reduction of 90%, the starting rolling temperature and finishing rolling temperature are 1050 °C and 950 °C, respectively. Finally, the hot rolled strip was cold rolled to 2 mm at a rolling reduction of 44.4%. Manganese was used to improve the stability of austenite, while silicon was added to restrain the formation of cementite. Furthermore, both elements were effective for improvement of the steel’s uniform elongation and formability [16,17]. Vanadium was also added to introduce grain refinement and precipitation strengthening [18]. A dilatometry experiment (DIL805A, BAHR-Thermoanalyse GmbH, Hullhorst, Germany) (see Figure 1a) was firstly conducted to measure the phase transformation temperature *Ac1* (ferrite-cementite to ferrite-austenite point), *Ac3* (ferrite-austenite to austenite point) and *Ms* (martensite transformation starting temperature), which were 750 °C, 910 °C and 330 °C, respectively.

Specimens of 2 × 25 × 120 mm^3^ cut from cold rolled strip were heat treated in a continuous annealing simulating testing instrument (CCT-AY-II, Ulvac-Riko INC., Tokyo, Japan) under the optimized heat treatment process as shown in Figure 1b. The intercritical annealing (IA) temperature was determined as the value near 1/2(*Ac1* + *Ac3*) to achieve the optimum *γ/α* ratio (approximately 50% *γ* + 50% *α*) with preferable mechanical properties [19]. Isothermal holding temperature was chosen between *Ms* and *Bs* where isothermal bainite transformation (IBT) would proceed without martensite transformation. Sufficient isothermal holding time was necessary for bainite transformation and carbon partitioning [11]. Black solid line represents the heat treatment process, samples were annealed at 850 °C for 120 s and isothermal held at 380 °C for 400 s before cooling down to ambient temperature, the heating rate and cooling rate were 10 °C/s and −50 °C/s, respectively. Red solid line represents dilation of the tested specimen, two horizontal steps durining IA and IBT indicate the completion of austenization and bainite transformation, respectively.

### 2.2. Tensile Tests and Microscopy Characterization

Dog-bone shaped specimens cut from the heat treated strip for tensile tests were prepared parallel to the rolling direction with a gauge section of 15 × 5 × 2 mm^3^. Uniaxial tensile experiments were performed on a WDW-2000D universal testing machine (Shanghai Xieqiang Instrument Technology Co., Ltd., Shanghai, China) at a strain rate of 10^−3^ s^−1^. The specimens were deformed to specific strain levels, i.e., 0%, 5%, 10%, 15%, and 20%.

Metallographic observation and analysis were all performed on the planes parallel to the transverse direction (TD) and rolling direction (RD). Specimens for optical microscope (OM) (Carl Zeiss Microscopy GmbH, Jena, Germany) and scanning electron microscope (SEM) (Zeiss Ltd., Braunschweig, Germany) were machined and mechanically polished, and then LePera reagent for OM and 5 vol% nital etchant for SEM were used to reveal the microstructure components. Specimens of 0.6 mm in thickness for transmission electron microscope (TEM) (JEOL Ltd., Tokyo, Japan) were mechanically polished to 40 μm and punched into Φ3 mm discs, and then they were twin-jet electro-polished in 5 vol% perchlorate alcohol solution at 50 V/−30 °C for about 2 min.

Specimens of 2 mm in thickness for X-ray diffraction (XRD) and electron backscatter diffraction (EBSD) were mechanically polished, and then they were electro-polished in 5 vol% perchlorate alcohol solution at 36 V/20 °C for 10 s. XRD measurements were performed over a 2θ degree ranging from 47° to 93° with a step size of 0.02° and a scanning speed of 1°/min in Rigaku D_MAX_-RB X-rays diffractometer (Rigaku Corporation, Tokyo, Japan). Volume fraction of RA (*V_γ_*) and its carbon content (*C_γ_*) were calculated from the integrated intensities of (200)*_γ_*, (220)*_γ_*, (311)*_γ_* peaks of fcc phase and (200)*_α_*, (211)*_α_* peaks of bcc phase [3,20,21]. EBSD experiments were performed at a step size of 50nm using a Zeiss ULREA 55-type field emission electron microscopy (Zeiss ULREA 55-type, Oberkochen, Germanny) equipped with an HKL Nirdlys F+ probe (OXFORD Instruments, Oxford, UK). 

## 3. Results and Discussion

### 3.1. Microscopy and Mechanical Properties after Heat Treatment

#### 3.1.1. Microstructure Components and Tensile Properties

**Figure 2:**
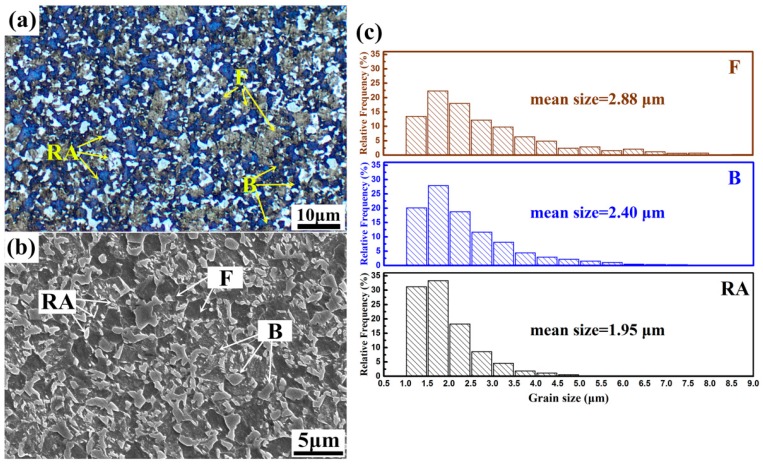
Micrographs of the heat treated specimen (**a**) color metallographic OM micrograph by LePera etching (**b**) SEM secondary electron image and (**c**) grain size statistics of different microstructure components by image analysis software based on color metallographic OM micrographs.

Figure 2 shows microstructure components of the experimental steel after heat treatment process, which consists of ferrite (F), bainite (B), and retained austenite (RA). Color metallographic OM micrograph shown in Figure 2a reveals ferrite (in grey), retained austenite (in white) and bainite (in blue). Microstructural analysis by image processing software indicates the constituents of the steel with approximate 15 vol% RA, 39 vol% B, and 46 vol% F. A very fine mean grain size (see Figure 2c) with *d_RA_* = 1.95 μm, *d_B_* = 2.40 μm and *d_F_* = 2.88 μm is also measured in different microscopes. SEM micrograph shown in Figure 2b reveals that RA mainly locates in ferrite grain boundaries and distributes in bainite matrix, most of the RA exhibits blocky-type morphology. Tensile tests reveal that the average yield strength (YS), ultimate tensile strength (UTS), uniform elongation (UEL) and total elongation (TEL) of the steel are 689 ± 15 MPa, 1106 ± 10 MPa, 20.4 ± 0.5% and 27.7 ± 0.8%, respectively. Therefore, the value of PSE is 30.6 GPa·%. The extraordinary mechanical properties of the steel might be achieved by the TRIP effect, precipitation hardening, grain refinement and dislocation strengthening, which will be discussed subsequently.

#### 3.1.2. RA Morphology and Distribution

**Figure 3:**
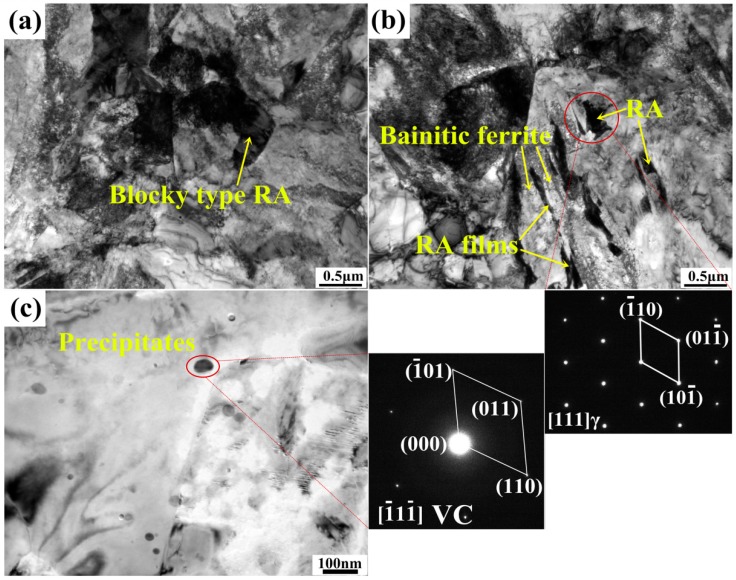
TEM micrographs of the heat treated specimen (**a**) RA located on grain boundaries (**b**) RA distributing between bainitic ferrite (**c**) precipitates in ferrite grain.

TEM micrographs as shown in Figure 3 reveal the detailed microstructure of the steel. Blocky-type RA on grain boundaries (see Figure 3a) and film-like RA between bainitic ferrite (BF) (see Figure 3b) are most commonly seen, numerous vanadium carbides (see Figure 3c) that widely disperse in ferrite grains are also observed. RA blocks on grain boundaries exhibit an average size of 1–2 μm, while RA films separated by BF plates exhibit a multi-scale microstructure with an average size of 50–100 nm in thickness and 0.5–2 μm in length. It is researched that blocky-type RA usually contains various defects and shows less stability, such a type of RA will easily transform into martensite during straining [22]. As for the film-like RA, it usually shows higher stability. This is mostly because the high hydrostatic pressure of the adjacent BF plates that leads to improved mechanical stability and lower strain concentration in the RA films [11]. The morphology effect and surrounding microstructure play significant roles for the RA stability in spite of carbon content. It is researched that a low carbon (0.64 wt%) film-like austenite is mechanically stable up to a strain of 12%, while the high carbon (1.14 wt%) blocky austenite transforms into twin martensite at the onset of plastic deformation [23], indicating the better stability of film-like RA than blocky-type RA. Thus, the blocky-type RA tends to transform at early deformation stage, while the film-like RA with appropriate stability tends to a gradual transformation throughout continuous straining [6].

### 3.2. RA Transformation during Deformation

#### 3.2.1. RA Transformation during Deformation

**Figure 4:**
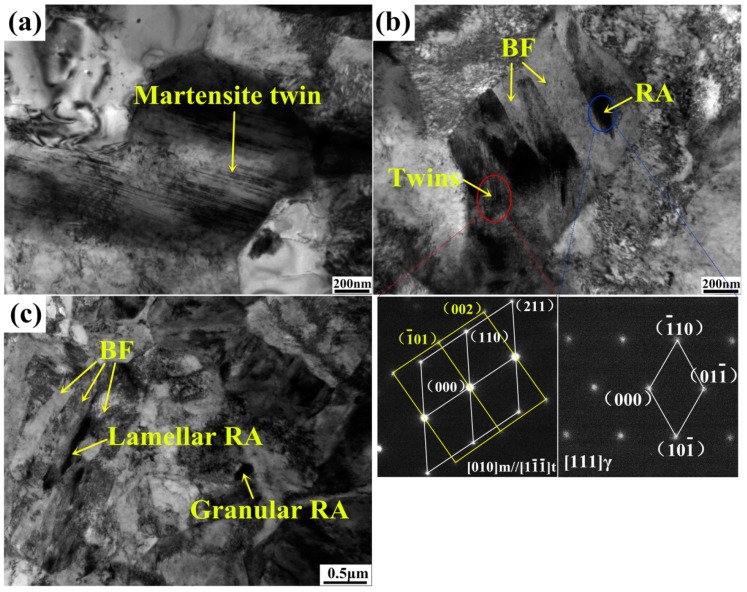
TEM micrographs showing RA and martensite morphology after deformation: (**a**) blocky-type RA transformation to martensite twin at 15% strain; (**b**) film-like RA transformation to martensite twin at 15% strain; (**c**) untransformed RA between bainitc ferrite plates and in ferrite grains observed in fractured tensile specimen.

Figure 4 shows martensite twins and RA transformation procedure during plastic deformation. The martensite twins are widely seen in the matrix, and most of blocky-type RA (see Figure 4a) transforms into martensite twins after 5% strain. For the film-like RA (see Figure 4b) distributing between BF plates, only a part of these RA films transforms and the rest part of them does not transform after 15% strain. Additionally, there is still film-like RA and some granular RA that existed in the fractured specimen (see Figure 4c) while no blocky-type RA was observed. The RA evolution during plastic deformation indicates that film-like RA in the experimental steel exhibits better mechanical stability compared to blocky-type RA. The difference of RA stability is a result of morphology effect, grain size effect and also distinct carbon content. As for RA with smaller grain size, higher elastic strain energy is needed for austenite to martensite transformation, which means decreasing in grain size will put off martensite nucleation and improve RA stability [3]. In addition, energy spectrum analysis indicates higher carbon content in film-like and granular RA compared to the blocky one. Therefore, the blocky-type RA shows easier transformation behavior at early deformation stage, while the film-like RA exhibits gradual transformation throughout deformation.

**Figure 5:**
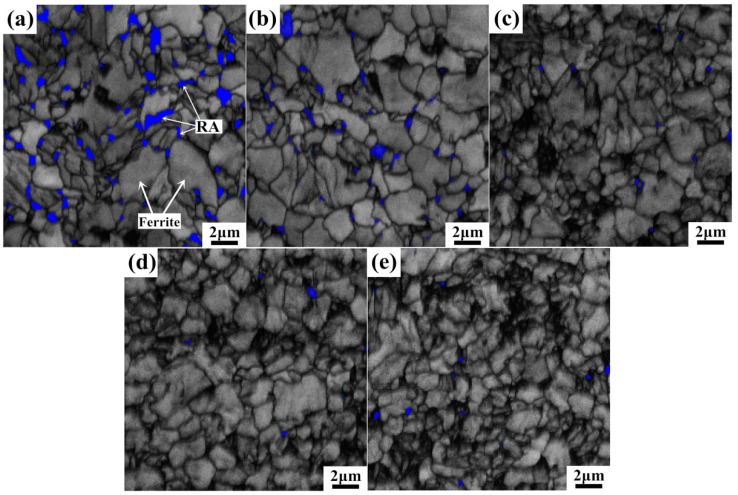
Representative EBSD maps showing face-centered cubic (FCC) phase (RA) and body-centered cubic (BCC) phase (ferrite, martensite, bainite) at different strain levels: (**a**) 0%, (**b**) 5%, (**c**) 10%, (**d**) 15%, (**e**) 20% (RA in blue, BCC phase in grey, high angle (>15°) grain boundaries in thick black lines, and low angle (<5°) grain boundaries in thin black lines).

EBSD maps shown in Figure 5 reveal the microstructure evolution at incremental straining. It is apparent to see that the blocky-type RA on ferrite grain boundaries easily transforms at lower strain levels, the majority of RA blocks already transform to bcc phase at 10% strain level and only a small number of granular RA is retained at 10%, 15%, and 20% strain levels. Lager RA blocks exhibit easier transformation ability while the small granular ones usually possessed higher carbon content and higher mechanical stability so as to be preserved throughout deformation. These retained granular RA is mostly located on the three-point conjunctions of bcc grains, and the high mechanical stability makes them become effective strengthening phase of the steel.

Dark areas are where mechanically induced martensite locates. Volume expansion caused by martensite transformation needs to be accommodated by surrounding soft ferrite. This leads to the generation of dislocations in the newly formed martensite and in the adjacent ferrite grains. As a result, dislocation accumulation to accommodate strain gradient is occurring with increasing strain levels. It is also observed that low angle grain boundaries exhibit rapid growth over 10% straining, initial ferrite grains are divided into smaller substructures during further deformation. This phenomenon is mostly controlled by the dislocation configurations. As the majority of RA component already transforms at early deformation stage, dislocation interactions in ferrite undertake a significant role for plastic deformation of the steel. The high density dislocations formed at high strain levels will arrange themselves into more stable cell structures and lead to the formation of substructures in ferrite grains.

**Figure 6:**
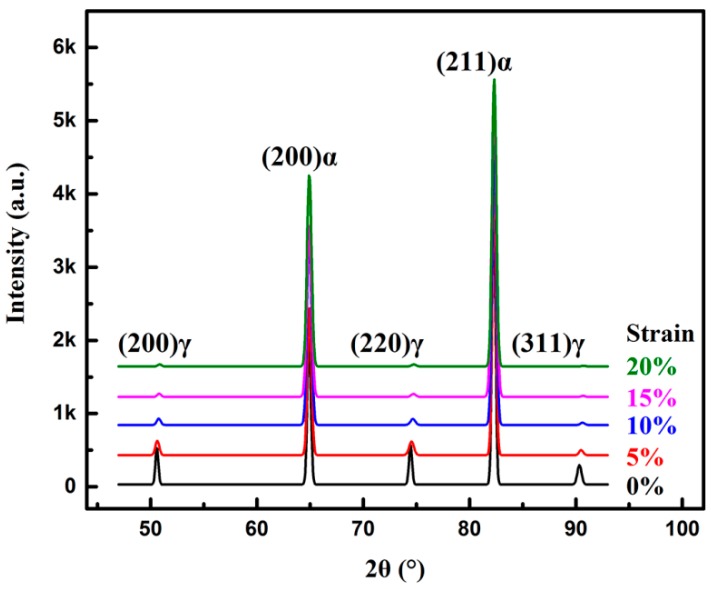
X-ray diffraction spectra of the specimens at different strain levels ranging from 0% to 20% in the strain rate of 0.001 s^−1^.

Figure 6 shows the X-ray diffraction spectra of the specimens at incremental strain levels. The (200)*_γ_*, (220)*_γ_*, (311)*_γ_* peaks of fcc phase (retained austenite) exhibit a decreasing tendency with increasing strain levels, indicating the consumption of RA due to the TRIP effect. Before tensile experiment, the initial RA volume fraction is 14.4 ± 0.7% with average carbon content 1.16 wt%. At 10% and higher strain levels, peak intensities of the fcc phase are very weak, which means the RA volume fraction reaches a very low degree at the strain level of higher than 10%.

#### 3.2.2. Relationship between RA Volume Fraction and Strain Hardening Rate

Table 1 lists the calculated RA volume fraction by XRD and EBSD at different strain levels. Both measurements indicate that more than 77% of total RA transforms at the strain level 5% and less than 10% of total RA remains at the strain level of 10%. This is a result of a high proportion of blocky-type RA, which has low stability that tends to transform at early plastic deformation.

**Figure 7:**
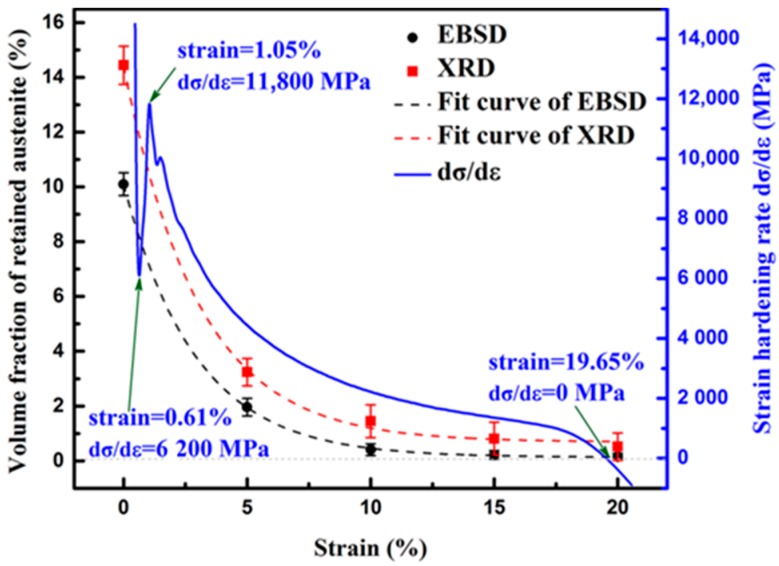
Variation curves of RA volume fraction and strain hardening rate with increasing strain.

Figure 7 portrays the fitted variation curves (in black and red dash lines) of retained austenite volume fraction based on XRD and EBSD results. Both fitted curves accord with exponential relationship which can be described as:

(1) Vγ=Ae−Rε

where *V_γ_* is the volume fraction of RA, *ε* the strain level, *A* and *R* the positive constants. As the curves and equations reveal, *V_γ_* decreases from fast to slow with increasing strain *ε*. When specimens deform at the strain level of 10%, nearly 90% of RA has transformed already and the decreasing tendency begin to slow down.

Strain hardening rate (d*σ*/d*ε*) variation curve (starting at strain = 0.37%) is also plotted in Figure 7. The d*σ*/d*ε* curve exhibits a continuous strain hardening characteristics during uniform plastic deformation. The upward curvature of the curve at the initial straining stage over strain = 0.61% indicates the occurrence of accelerated strain hardening during plastic deformation. It reaches the maximum value of 11,800 MPa with strain = 1.05%. The fast growth of strain hardening occurs during yielding stage. This is because the dislocation gliding is retarded by the pinning effect of the vanadium carbides and also the fast transformation of metastable RA blocks that contribute to reinforcement of the steel matrix, which effectively promotes strain hardening at the yielding stage. During the uniform elongation ranging from 1.05% to 19.65%, the strain hardening rate exhibits a continuous exponential decreasing tendency, which is in accordance with the fitted curve of the RA consumption. It means that the RA component plays a significant role for strain hardening of the steel, the rapid transformation of blocky-type RA ensures good strain hardening ability at the early deformation stage, film-like RA with improved mechanical stability exhibits continuous transformation and sustains the continuous strain hardening during plastic deformation.

### 3.3. Substructure Formation and Ferrite Grain Subdivision under Strain

#### 3.3.1. TEM Observation of Dislocation Evolution and Ferrite Subdivision

**Figure 8:**
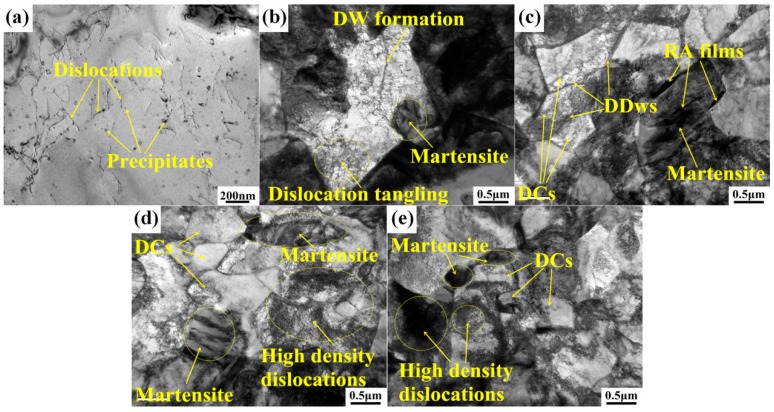
TEM micrographs of dislocations and precipitates in ferrite before deformation: (**a**) precipitates and dislocations in ferrite. Substructures formation procedure accompanied with growing dislocation density in ferrite at incremental strain levels: (**b**) 5%, (**c**) 10%, (**d**) 15%, and (**e**) 20%.

TEM micrograph as shown in Figure 8 reveals the dislocation evolution and substructure formation during tensile straining. Relatively high density dislocations (see Figure 8a) and a certain amount of precipitates are observed in ferrite grains before deformation. The original dislocations are the result of bainite transformation and fast cooling rate during heat treatment. Higher flow stress is needed with higher dislocation density according to the Taylor relationship [24], the nanoscale precipitates (mainly vanadium carbides with diameter of 10~50 nm in bcc grains) can also effectively retard the migration of newly formed dislocations [18], resulting in enhanced deformation resistance of the steel.

Figure 8b shows the formation of dislocation tangles and dislocation walls (DWs) at the strain level of 5% in a ferrite grain. Strain accumulation on grain boundaries and volume expansion caused by martensite transformation lead to some initial plastic deformation in ferrite grains, resulting in the generation of dislocations at the adjacent ferrite interface. The original dislocations have high three-dimensional mobile ability and will easily transform to cellular substructures at higher strain amplitude due to the multiple slip system activity of bcc phase [25]. The active dislocation movement and precipitate fixing effect cause effective dislocation interactions, leading to sustaining pile-ups and accelerating formation of DWs in the early stage during deformation [26,27].

Owing to the strain accumulation during straining, dislocations will gradually arrange themselves into relatively more stable configurations, i.e., the dislocation cells (DCs) [12,13,28]. Figure 8c shows that the DCs formation in coarse ferrite grain with 10% strain. Ferrite grain is subdivided into several DCs delimited by sharp dislocation boundaries. These dislocation boundaries are the dense dislocation walls (DDWs) that act as geometrically necessary boundaries between two adjacent DCs of different orientation. Sizes of DCs are mostly in the range of 0.5~1 μm. Ferrite grains are divided by these DCs, leading to the so called ferrite subdivision. It leads to grain refinement and ferrite reinforcement according to the so called Hall-Petch law [29,30]. The contribution to the flow stress by dislocation substructure evolution can be evaluated by widely used empirical relationship [12,13]:

(2) σ=Mτ=MGKb/D

where *M* is the Taylor factor, *G* the shear modulus (~70 GPa for austenite and ~80 GPa for ferrite), *b* the Burgers vector, *D* the substructure size and *K* the constant. Equation (2) indicates that the formation of dislocation substructures raises the flow stress during straining and, thus, strengthens the steel matrix.

With strain continuously increases to 15% (see Figure 8d) and 20% (see Figure 8e), more and more fresh dislocations form on grain boundaries or within ferrite substructures and massive DCs with DDWs as grain boundaries are produced. The formations of DCs and the high density dislocations facilitate considerable strain energy absorbing, and play important roles for further improved mechanical properties of the steel. Dislocation gliding and interactions are limited in these sub-structural areas, and the steel is highly strengthened by the refined ferrite grains with a highly accumulated strain level.

#### 3.3.2. EBSD Investigation of Local Misorientations in Ferrite

**Figure 9:**
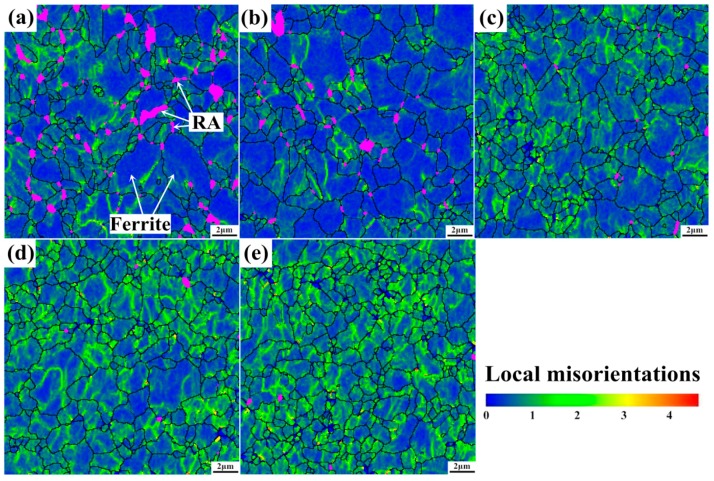
Local misorientation distribution maps of BCC phase of tensile tested specimens with different strain level: (**a**) 0%, (**b**) 5%, (**c**) 10%, (**d**) 15%, and (**e**) 20% (BCC grain boundaries in dark solid lines and retained austenite grains in purple color areas).

Plastic deformation and local strain, which are physically associated with dislocation and crystal-lattice rotation, can be evaluated by misorientation distribution maps [31,32]. The misorientation (orientation gradients range from 0° to 5°) maps in bcc phase (ferrite, bainite, or martensite) are shown in Figure 9. Areas marked by rainbow color in bcc grains indicate the local misorientation distribution. It can be observed that the specimens of the undeformed (see Figure 9a) and the deformed with 5% plastic strain (see Figure 9b) exhibit similar local misoriention distribution and bcc grain size. This can be explained by the sharply declined RA (in purple color) at initial straining, which plays an important role in absorbing strain energy to accommodate local strain gradient. When strained at 10% (see Figure 9c), rainbow areas ranging from 2° to 3° show dramatic increase in bcc grains. As mechanical induced martensite transformation of RA, which is very effective for strain energy absorbing and strain accommodation, has almost completed. Thus, the bcc grains have to undertake much more strain and stress at higher strain levels, leading to increase of dislocation density in bcc grains and also increase of rainbow areas in comparison with strain level of 5%. Moreover, misorientations of linear morphology subdivide the bcc grain into several blocks, just in accordance with dislocation cellular substructures as observed in Figure 8, because the DCs are delimited by the geometrical necessary boundaries (DDWs) for accommodating the lattice misorientation between neighboring substructures. Additionally, local strain hardening caused by the TRIP phenomenon makes the flow path of ferrite inhomogeneous and leads to dislocation accumulation to accommodate strain and stress gradient [33].

When strain increases to 15% and 20% (see Figure 9d,e), some rather high angle (2°~5°) misorientations (orange or red plots) arise mostly along with the grain boundaries. Dislocation density in bcc grains has reached to rather high level, the active dislocation movement and precipitate fixing effect lead to effective dislocation interactions, and high density dislocation walls begin to form in bcc grains, resulting in constrained dislocation movement. Moderate lattice curvature or lattice rotation begins to occur for strain accommodation. As a result, rather high angle misorientations occur in bcc grains and along with the grain boundaries. A large increase of rainbow lines indicates DCs formation in bcc phase, resulting in the subdivision and refinement of bcc grains.

**Figure 10:**
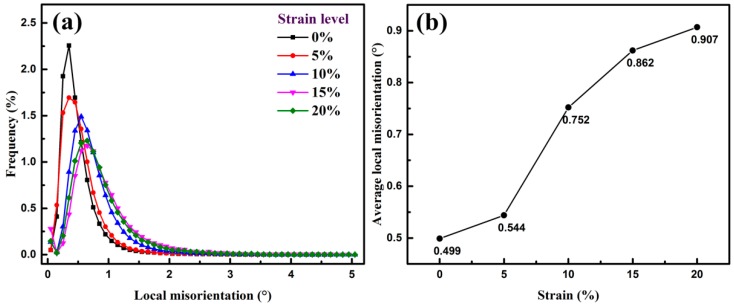
Change of (**a**) local misorientations and (**b**) average local misorientations of BCC phase at different strain levels.

Figure 10a shows the variation curve of low angle (0°~5°) local misorientations of the bcc phase. It can be noted that the local misorientation curve with maximum frequency gradually increases and the peak width broadens with increasing strain. Average local misorientation (0°~5°) plotted in Figure 10b exhibits a rising tendency with increasing strain ranging from 0% to 20%. It can be seen that average local misorientation go up slightly at the very beginning of deformation, while it increases rapidly from 0.544° straining at 5% to 0.752° straining at 10%. Then it keeps up growing with further deformation, whereas the incremental speed gradually slows down when strained from 10% to 20%. The misorientation variation tendency is in accordance with RA transformation and the dislocation sub-structures formation. RA possesses greater strain hardening ability than ferrite because of mechanically-induced martensite transformation. Most of the strain is accumulated in RA at lower strain level and, therefore, the local strain slightly increases in ferrite. The majority of RA in the steel exhibits blocky-type morphology and less stability, resulting in higher transformation speed at early deformation stage lower than 5%. Dislocation configuration and substructure formation in ferrite grains will then speed up during straining ranging from 5% to 10%. The decreased growth rate of average local misorientation is indicative of the high strain concentration by further plastic deformation over 10% strain. This phenomenon can be explained as a result of the increasing dislocation density and massive DCs, which limit further migration of the dislocations and, thus, improve the strength of the steel.

### 3.4. Microstructural Development during Deformation in the Multiphase Steel

**Figure 11:**
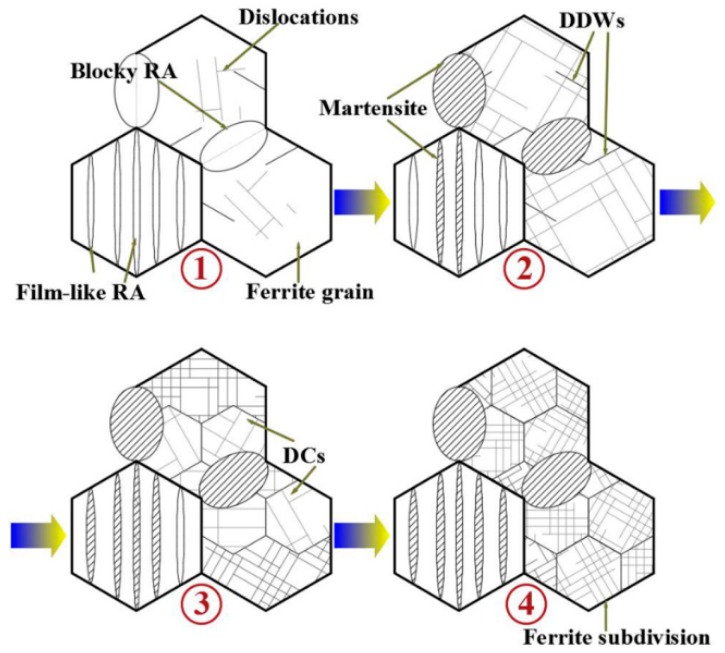
Schematic figure of the microstructure evolution during deformation of the tested steel.

Figure 11 illustrates the collaborative deformation-transformation behavior and microstructure evolution of the steel during plastic deformation. The experimental TRIP-assisted steel exhibits a mixture of ferrite, bainite, and retained austenite. Plastic deformation contributes to strain accommodation in each constituent phase, which first begins at the yielding stage. During the subsequent deformation, austenite exhibits the largest strain, followed by bainitic ferrite, and that of the ferrite matrix is the smallest [34]. The blocky-type RA located on ferrite grain boundaries is less stable compared to the film-like RA distributed between BF plates, and it quickly transforms into martensite at the early plastic deformation stage. The film-like RA transformation between bainite plates is controlled by dislocation slip deformation, which increases local stress at austenite-bainite interface and leads to reinforced mechanical stability of RA. As a result, RA blocks tend to transform during early deformation while the film-like RA exhibits gradual transformation throughout deformation. RA consumption is fast at low strain levels because of high blocky-type RA proportion of the total RA constituent in the present multiphase steel, resulting in high strain hardening rate at low strain levels. The TRIP-assisted collaborative deformation comes to an end at the point where the RA volume fraction decreasing rate becomes zero and necking starts.

The macroscopic yielding of the steel takes place through the simultaneous cooperative activity of the austenite-to-martensite transformation in the austenite phase and plastic deformation in the ferrite matrix [35,36]. In the multiphase steel, the soft ferrite offers necessary plasticity, and the plastic deformation in ferrite is governed by dislocation gliding. The gradient of the plastic deformation in ferrite at different strain levels can be accommodated by the geometrically necessary dislocations. With a considerable of nanoscale precipitates widely dispread in the ferrite matrix, massive dislocations tangling occur in interior ferrite grains by precipitates pining effect. Moreover, dislocations configuration in ferrite begins with randomly distributed dislocations at low strain level and ends up with DCs at higher strain level, leading to ferrite subdivision. The continuous formation of DCs with increasing strain is effective to enhance the soft ferrite matrix, so as to undertake larger stress and strain component of the constituent phases.

## 4. Conclusions

Fine-grained multiphase steel with a good combination of high strength and ductility is produced. Microstructure evolutions of RA transformation, dislocation configuration, and ferrite subdivision during deformation are investigated. The conclusions of this work are as follows:

(1) The multiphase steel exhibits a mixture of soft ferrite matrix, a strong bainitic phase, a suitable proportion of 14.4 vol% RA and numerous vanadium precipitates. The steel exhibits fine-grains (<5 μm) with high UTS 1106 ± 10 MPa and TEL 27.7 ± 0.8%, and the PSE 30.6 GPa·%.
(2) RA transformation is mainly controlled by its morphology and distribution. RA in this steel is mostly blocky-type that distributes on ferrite grain boundaries and will easily transform at the beginning of straining because of its low stability. Film-like RA that distributes between BF plates exhibits gradual transformation tendency owing to its higher carbon content, smaller grain size and surrounding BF structures, which lead to higher mechanical stability of the film-like RA.
(3) The change of retained austenite volume fraction can be described as *V_γ_ = Ae*^−*Rε*^ in exponential relationship, which indicates the fast transformation rate at the early deformation stage and rather gentle variation tendency at higher strain level. The fast transformation of blocky-type RA contributes to relatively high strain hardening rate during early deformation and the gradual transformation of film-like RA contributes to continuous strain hardening throughout uniform plastic deformation.
(4) Continuous dislocation configuration in ferrite begins with randomly distributed dislocations and ends up with DCs through the whole plastic deformation, leading to grain subdivision and refinement strengthening of ferrite phase. Ferrite subdivision caused by dislocation substructure evolution associated with precipitating strengthening plays vital roles for further improving strength of the steel, which is effective to suppress onset of necking to achieve lager uniform elongation.

## Figures and Tables

**Table 1:** Volume fractions of retained austenite at different strain levels measured by XRD and EBSD.

Strain (%)	Volume Fraction of Retained Austenite (%)	
	XRD Results	EBSD Results
0	14.4 ± 0.7	10.12 ± 0.42
5	3.2 ± 0.5	1.96 ± 0.32
10	1.4 ± 0.6	0.41 ± 0.21
15	0.8 ± 0.6	0.18 ± 0.15
20	0.7 ± 0.5	0.15 ± 0.12
